# Metastatic Basal Cell Carcinoma With Unusual Molecular Genetics Findings

**DOI:** 10.1002/ccr3.71490

**Published:** 2026-04-12

**Authors:** Noora Al‐Hail, Majid Al‐Abdulla, Syed Rizvi, Nazeeha Al‐Hayki

**Affiliations:** ^1^ Department of Otolaryngology–Head and Neck Surgery Hamad Medical Corporation Doha Qatar; ^2^ Department of Otorhinolaryngology–Head and Neck Surgery Hamad Medical Corporation Doha Qatar; ^3^ Department of Laboratory Medicine and Pathology Hamad Medical Corporation Doha Qatar; ^4^ Department of Dermatology and Oncology Hamad Medical Corporation Doha Qatar

**Keywords:** basal cell carcinoma, lymphatic metastasis, molecular genetics, skin neoplasms

## Abstract

Basal cell carcinoma (BCC) rarely metastasizes to other parts of the body. When metastasis occurs, it usually involves regional lymph nodes. BCC lesions located in the head and neck region have been shown to spread to cervical lymph nodes. Here, we present a case of a slow‐growing lesion on the scalp that was diagnosed as a case of BCC. Metastasis to the level V cervical lymph nodes was already present at the time of diagnosis. The patient was managed with complete excision of the lesion, along with a neck dissection for level V lymph nodes, and adjuvant chemotherapy with Vismodegib. Molecular pathology results revealed a unique pattern, comprising the fusion of HMGA2‐CHMP1A genes and a mutation involving a premature termination codon (PTC) in the CDKN2B gene. The post‐operative work‐up revealed a hypermetabolic nodule in level IIB lymph which is more likely inflammatory rather than metastatic. However, considering the possibility of metastasis, the patient is being followed up by a multi‐disciplinary team (MDT) for evaluation of level IIB cervical lymph nodes. This case highlights the diagnostic and therapeutic challenges that may arise in rare cases of BCC with metastasis.

**Summary**

- Presented tumor exhibited HMGA2‐CHMP1A gene fusion, a CDKN2B gene mutation (premature termination codon at R82Ter) and a loss of p53.
- While mutations in PTCH1, SMO, and TP53 are commonly implicated in BCC, this is the first reported case involving CDKN2B and HMGA2‐CHMP1A fusion in metastatic BCC.
- These genetic alterations may contribute to tumor progression and metastatic potential.

## 1 Introduction

Basal cell carcinoma (BCC) is the most common skin cancer in fair‐skinned individuals. The lifetime risk in these individuals is estimated to be as high as 30% [1]. The highest incidence of BCC is in Australia and New Zealand, where the incidence of BCC is more than 1000 per 100,000 of the population [2]. The global incidence of BCC is on the rise, and there is a projected yearly increase of 5% in the incidence of BCC in Europe and more than 2% in the United States [3]. The incidence in the Asian populations is 10–100 folds lower than that of the Western countries [3]. As per statistics from the year 2020, the age‐specific incidence rate (ASIR) of various types of cancers in the population of Saudi Arabia is 89 per 100,000 population [4]. Skin cancer is not very common in the Saudi population; nevertheless, among patients with skin cancer, BCC and squamous cell carcinoma (SCC) form the majority [5].

The development of BCC is dependent on various genetic and environmental factors. High exposure to ultraviolet (UV) radiation is a major cause of the development of BCC. In addition to UV radiation, occupational or iatrogenic exposure to ionizing radiation is also believed to be a cause of BCC [6]. Males and fair‐skinned individuals are at higher risk of developing BCC, particularly those with skin types that are more susceptible to sunburns, such as Fitzpatrick type I or II skin [7]. Most BCCs are confined to the skin, particularly in the exposed parts of the body such as the head and neck region. The incidence of metastasis in BCC is very rare and ranges from 0.0028% to 0.55% [8]. The most common route of metastasis is through lymphatic spread to regional lymph nodes, followed by hematogenous spread to the lungs or bones in some cases. Cases of BCC with metastasis have a much poorer prognosis, and generally, there are no clear‐cut guidelines on the management of metastatic BCC [9].

Metastasis in a BCC has been shown to occur in large, longstanding lesions, which show ulceration and are histologically aggressive tumors [10]. The majority of sporadic BCCs show loss of heterozygosity of the PTCH1 gene or inactivating TP53 alterations along with mutations in TP53, SUFU, MYCN, PPP6C, and STK19 genes, etc. [11]. As cases of metastatic BCC are very rare, there is limited data on the genetic mutations involved in their pathogenesis. Vismodegib is a Hedgehog signaling pathway inhibitor which is used for the treatment of BCC. Vismodegib‐resistant SMO mutation has been found to be involved in metastatic BCC [12]. Additionally, mutations in RET and HGF genes and phosphatidylinositol 3‐kinase (PI3K)/protein kinase B (AKT) signaling pathway are also believed to be involved in metastatic BCC [13]. Although there is substantial evidence on the role of HMGA2, CHMP1A and CDKN2B genes in the development of various skin cancers [12, 14], their specific role in the development of metastatic BCC has not previously been described. In this case report, the authors describe unusual molecular genetics in a case of metastatic BCC who has been under treatment for her metastatic disease.

## 2 Case History

**FIGURE 1:**
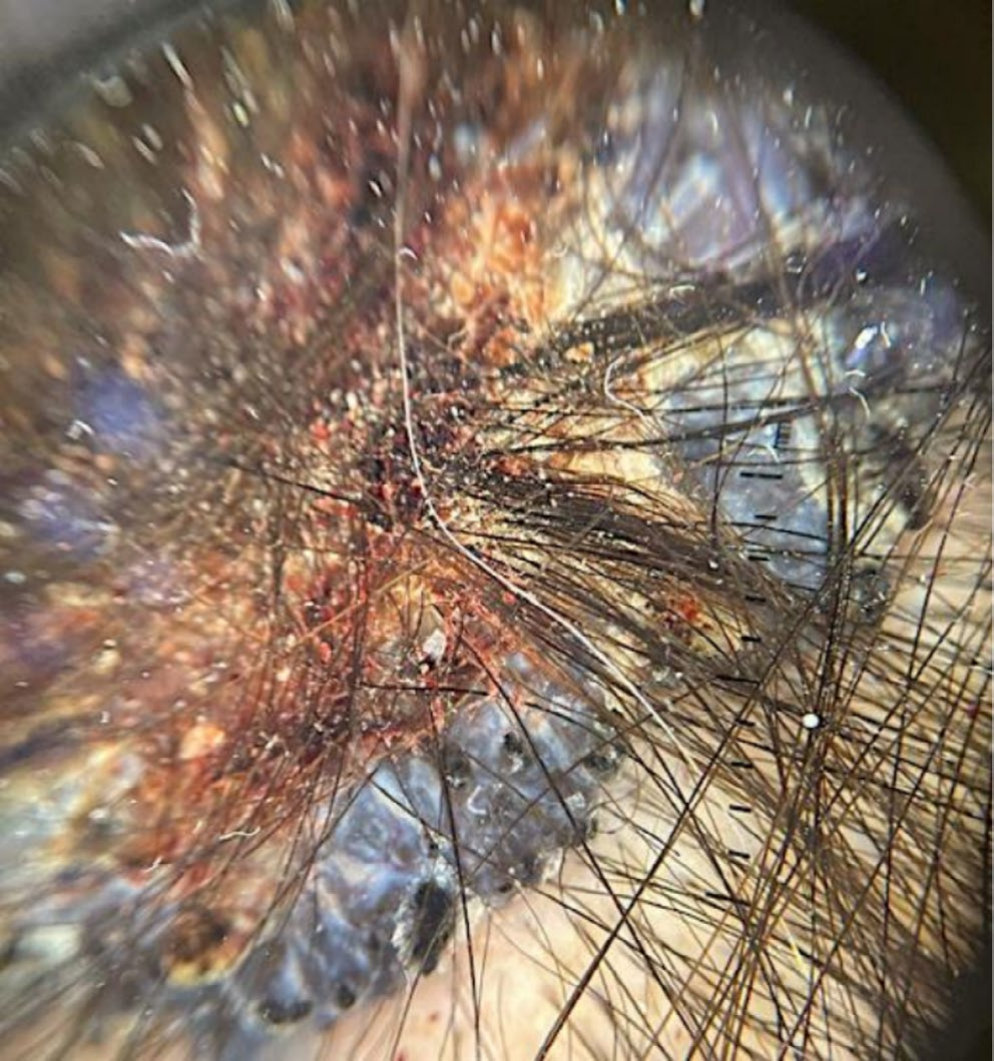
Dermoscopic image of the scalp lesion, revealing ulceration, prominent white streaks, and dense ovoid nests.

In September 2023, a 37‐year‐old female, with no prior medical problems, presented to the head and neck clinic with a progressively enlarging lesion on the right side of her scalp. She had first noticed the lesion 10 years back. The lesion gradually increased in size over the course of 10 years and started producing foul‐smelling discharge 1 month ago. On examination, the patient had a 3.5 × 3 cm plaque on the upper right side of the scalp with irregular margins, central ulceration, and crusts on the surface. The patient had lymphadenopathy in the right posterior triangle of the neck involving level V lymph nodes. She was referred to the dermatology clinic for dermoscopy and biopsy (Figure 1). The patient had no prior history of any other malignancy. However, she reported a family history of endometrial cancer in her mother. An ultrasound of the neck was performed, which confirmed suspicious cervical lymphadenopathy in the right upper posterolateral aspect of the neck. Ultrasonography of the thyroid demonstrated findings consistent with thyroiditis.

## 3 Methods

**FIGURE 2:**
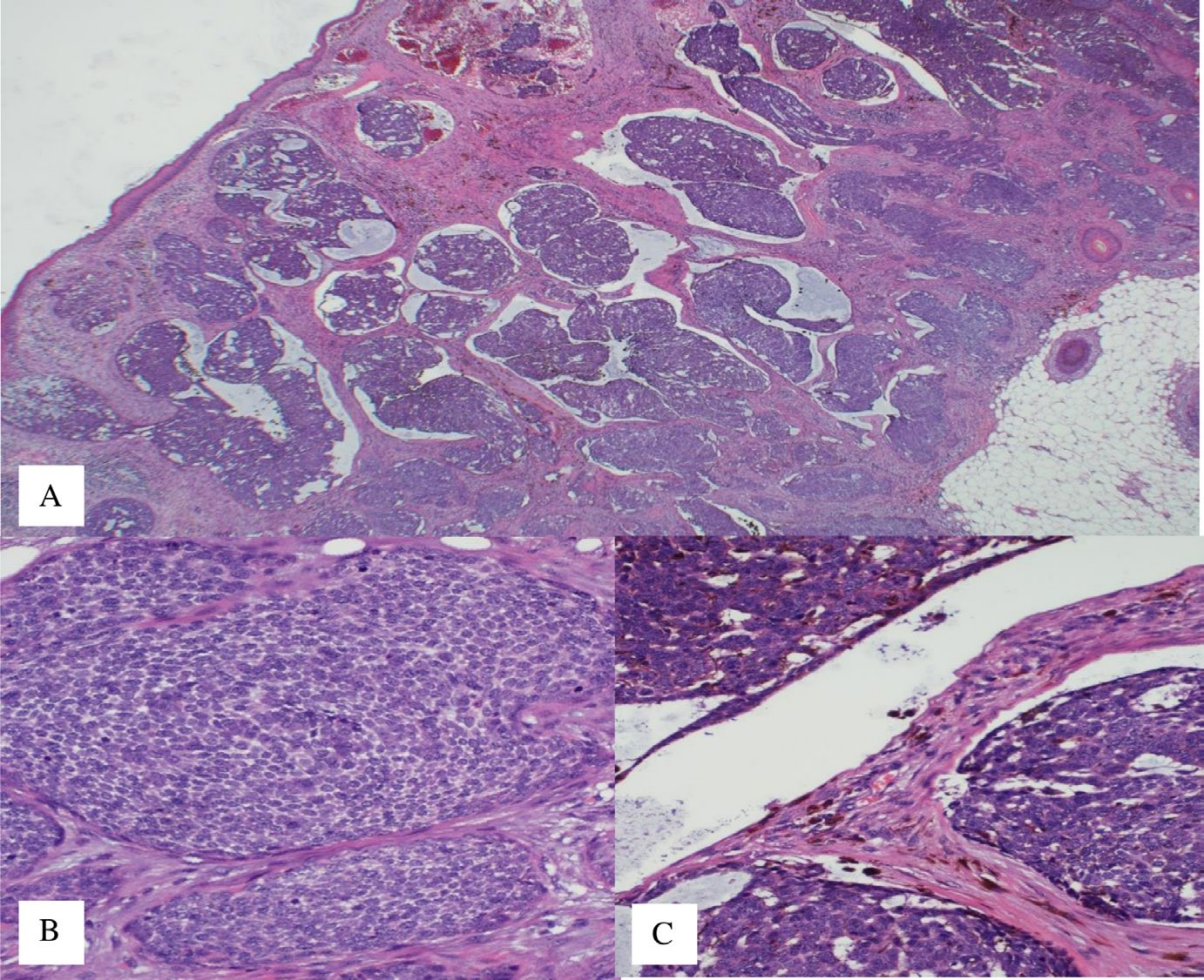
Histopathological findings showing a nodular and cystic pattern (A), with infiltration into the subcutis and solid nodules lacking prominent peripheral palisading (B). Additionally, areas exhibiting retraction artifact and pigmentation are observed (C).

**FIGURE 3:**
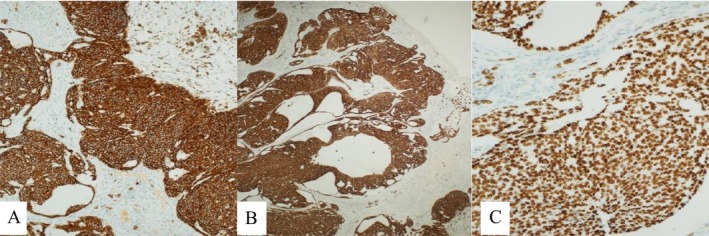
Primary tumor: Immunohistochemical staining revealed diffuse positivity for Ber‐EP4 (A), CK7 (B), and p40 (C), along with p63 and CK5/6. In contrast, EMA, CK20, Sox10, Melan‐A, Chromogranin, and Synaptophysin were negative.

**FIGURE 4:**
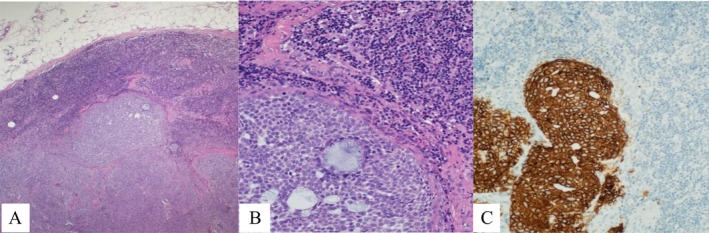
Metastatic tumor: (A) Low‐power view showing the rim of the lymph node and tumor. (B) Higher magnification highlighting tumor cells in the lower half and lymphocytes in the upper half. (C) Tumor cells demonstrating positive staining for Ber‐EP4.

After a complete dermatological examination, an incisional biopsy was undertaken, and the specimen was sent for histopathological examination. The histopathology report returned with a diagnosis of nodular and focally infiltrative basal cell carcinoma (Figures 2 and 3). The patient was referred to the ENT‐Head and Neck Department for wide local excision, which was performed along with an excisional biopsy of the level V cervical lymph node. The histopathology report confirmed a pT2, pigmented, nodular, and infiltrative basal cell carcinoma with focal ulceration. The tumor was completely excised. Molecular pathology results highlighted the fusion of HMGA2‐CHMP1A genes and a nonsense mutation involving a premature termination codon (PTC) in the CDKN2B gene at amino acid position 82 (R82Ter). The sampled lymph node revealed metastatic basal cell carcinoma (Figure 4).

Subsequent workup for metastasis was undertaken, and a CT scan of the neck with contrast highlighted suspiciously enlarged lymph nodes on the right side of the neck (level V) along with multiple other prominent bilateral cervical nodes with preserved fatty hilum. A Positron Emission Tomography/Computed Tomography scan (PET‐CT) and a Magnetic Resonance Imaging (MRI) scan of the abdomen were performed. The PET‐CT scan showed involvement of the right level V lymph nodes. Three liver lesions with features suggestive of hemangioma were reported on MRI. A diagnosis of metastatic BCC was confirmed at a multi‐disciplinary team (MDT) meeting discussion, and in the absence of distant metastasis, selective right neck dissection followed by adjuvant chemotherapy with Vismodegib was recommended.

At 1 month post‐operative, the scalp skin defect, which was closed using an O‐Z double rotational flap, had healed. The patient was informed about the MDT recommendation; however, she requested to seek a second opinion abroad regarding neck dissection and adjuvant chemotherapy (Vismodegib). The patient traveled to the United Kingdom (UK), and in May 2024, she underwent right‐sided selective neck dissection in the posterior triangle. Post‐surgery, the PET‐CT scan showed a small, mildly hypermetabolic, level IIB lymph node on the right side, which was more likely to be inflammatory rather than metastatic. Post‐procedural changes on the right side of the scalp were also reported.

## 4 Conclusion and Results

In a MDT meeting held in December, 2024, the patient was advised surveillance ultrasound of the neck to be conducted after 3 months. In case of progression of lymphadenopathy, a PET‐CT scan would be indicated. Considering the complexity of the clinical condition, close clinical follow‐up with multi‐disciplinary care will be continued until the patient is free of disease.

## 5 Discussion

Our report describes a case of metastatic BCC, which had spread from the primary lesion on the scalp to the ipsilateral cervical lymph nodes. The case demonstrates a unique molecular profile in the setting of metastatic BCC. Despite being the most common skin cancer, BCC has very low metastatic potential and an excellent prognosis if treatment is ensued in a timely manner [15]. However, there have been a number of cases of BCC that exhibited metastasis, thus posing a significant diagnostic and therapeutic challenge. The present case described a long‐standing lesion on the scalp that was slowly growing and did not prompt the patient to seek professional medical advice until she noticed a discharge from the lesion and the development of cervical lymphadenopathy. Delayed presentation can occur due to a number of reasons, such as a lesion away from the readily visible face or neck regions, absence of itching or bleeding in the lesion, no prior family history of skin cancer, and sometimes denial by the patient [16]. Delayed presentation in BCC is linked with an increase in size, the development of ulceration, and a higher probability of metastasis, all of which can significantly impact the patient's treatment and outcomes [17].

Various factors determine the prospects of metastasis in a BCC. The most important factors are the size, location and depth of the lesion. Lesions larger than 4 cm (OR: 11.9), located in the head/neck region (OR: 5.3), and involving tissues beyond skin fat (OR: 58.6) are primarily the factors responsible for the development of metastasis [18]. Our patient had a lesion approaching 4 cm that was located in the head/neck region and had a focally infiltrative morphology. Collectively, these features, along with a delayed presentation, raised the metastatic potential of the condition. The infiltrative type of tumor, as observed in this case, is characterized by abundant desmoplastic stroma with a high propensity for peri‐neural invasion and a higher rate of metastasis [19]. A case series involving six cases of metastatic BCC from Australia showed that most of the cases exhibited infiltration of the deeper layers of skin and it was recommended that particular attention should be paid to BCC showing infiltrative histology, specifically the lesions involving the head and neck region [20].

The site of metastasis observed in this patient was ipsilateral level V cervical lymph nodes along with a possible involvement of level IIB lymph nodes. Although the findings on PET‐CT scan are more consistent with inflammatory changes in level IIB lymph nodes, confirmation of the diagnosis would require further histopathological work‐up. Literature is evident that the most common route of spread of BCC is through the lymphatics to the regional lymph nodes, which accounts for more than 50% of the cases of metastatic BCC [21]. Metastasis to level II lymph nodes was observed in an Irish patient with BCC on right cheek [22] and in two cases of BCC of head and neck region reported in a study from Texas, USA [23]. The spread to the regional lymph nodes follows a predictable pattern. A coronal section of the scalp at a line joining the helix of ears separates the scalp into two halves. Tumors placed anteriorly metastasize to pre‐auricular, peri‐parotid, and anterior cervical lymph nodes of level I–IV, and posteriorly placed tumors spread to post‐auricular, occipital, and lymph nodes of the posterior triangle of the neck and along internal jugular vein [24]. Interestingly, the tumor in our patient was placed close to the watershed area and thus the spread to level V lymph nodes (posterior triangle of the neck) appears justified. A case series of 11 cases of metastatic BCC from Boston, USA, showed that regional lymph node metastasis was present in all these cases. However, interestingly only seven out of these 11 cases showed typical histological features of the primary lesion. In the remaining four cases, the morphology ranged from squamous differentiation to poorly differentiated tumor, thus raising the need for molecular testing to accurately arrive at the diagnosis [25].

**FIGURE 5:**
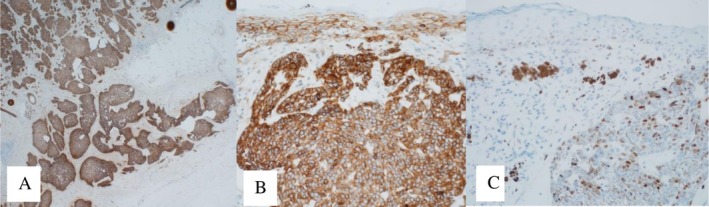
Beta catenin: Shows diffuse staining in the tumor (A) with a close‐up highlighting membrane/cytoplasmic staining (B) with no nuclear stain. P53 stain highlights the absence of staining in the overlying epidermis (C) with patchy staining in the primary tumor.

Given the limited number of metastatic BCC cases, the unpredictable behavior, and the lack of robust clinicopathological correlation with metastatic potential and therapeutic response, molecular testing is emerging as a key area of interest in predicting metastatic potential [26]. Most of the genes involved in the pathogenesis of BCC display mutagenic potential consistent with UV‐radiation‐induced damage to the DNA. The genetic mutations commonly observed in BCC include the PTCH1 gene that codes for the Sonic Hedgehog signaling pathway (SHH) [27]. Other commonly involved genes include the SMO gene, which codes for the Smoothened protein, which is another integral component of the SHH pathway. Tumor suppressor p53 protein is coded by the TP53 gene, the mutation of which, can lead to the loss of suppressor function of the p53 protein [28]. Mutation of the MYCN gene, a transcriptor gene commonly implicated in tumors such as neuroblastoma and retinoblastoma, is also regarded as a cause of BCC [11]. The eventual consequence of these mutations is uncontrolled growth of the cells in the basal layer of the epidermis, which leads to the development of BCC. Our patient exhibited loss of p53 staining in the overlying epidermis of the tumor (Figure 5).

Metastasis in a BCC is unpredictable and cannot be attributed to the mutation of a single gene. The most commonly identified genetic mutations in metastatic BCC include mutations in the PTCH1, SMO, TP53 and SUFU genes [12]. The results of genomic profiling conducted on 60 cases of metastatic BCC showed that, in contrast to BCC without metastasis, mutations in the TP53 gene are more common in metastatic BCC than in the PTCH1 and SMO genes [29]. However, in our patient, molecular testing revealed the fusion of HMGA2‐CHMP1A genes and a mutation in the CDKN2B gene. Although mutations in the CDKN2A gene have been found to be implicated in various skin cancers such as SCC and melanomas [30, 31], the authors could not explicitly find any previous evidence of CDKN2B mutation in reported cases of metastatic BCC. Nevertheless, there are similarities in both the CDKN2A and CDKN2B genes, as both act as cyclin‐dependent kinase inhibitors (CDKIs), although CDKN2A codes for p15 and CDKN2B codes for p16 tumor suppressive protein. The mutation of either of the CDKN2 genes can have a direct tumorigenic effect [32].

The other genetic defect observed in our patient was the fusion of HMGA2‐CHMP1A genes. The overexpression of HMGA2 has been shown to be linked with the development of various cancers as it binds to AT‐rich portions of DNA and alters the structure of chromatin [33]. To the contrary, CHMP1A is a member of the ESCRT‐III family of genes which regulate p53 expression and mTOR signaling pathway and thus has a role in the prevention of cancer [34]. The fusion of genes results in the loss of function of those genes, and if tumor suppressor genes are involved, then this fusion can result in tumorigenesis. The fusion of HMGA2 with other genes is a known mechanism for various tumors such as HMGA2‐EGFR fusion for brain tumors [35], HMGA2‐NCOR2 fusion for bone tumors [36], and HMGA2‐LPP or HMGA2‐GSN for lipomatous tumors [37, 38]. Mutation or fusion of CHMP1A can result in various cancers such as renal cell carcinoma [34], hepatocellular tumor [39], and pancreatic tumor [40]. Interestingly, CHMP1A is also known to affect the SHH pathway, which forms the basis of the development of BCC, thus pointing toward a possible role of CHMP1A mutation/fusion in the development of BCC [41]. Although no substantial literature evidence is present on the role of HMGA2‐CHMP1A fusion in BCC, nevertheless, the mechanism of action and implication of these genes in other tumors point toward a possible role of HMGA2‐CHMP1A fusion in the development of metastatic BCC. However, there is a dire need for further studies on the molecular genetics of metastatic BCC in order to fully understand the role of various genetic mutations in the development of this disease.

The prognosis of BCC without metastasis is excellent; however, with regional metastasis the mean survival is 87 months, which is further reduced to 24 months in cases of distant metastasis [42]. For this reason, a highly aggressive approach is adopted for the treatment of metastatic BCC. In our patient, wide local excision of the lesion was performed. After confirmation of lymph node metastasis, neck dissection was performed, and the patient was advised adjuvant chemotherapy with Vismodegib. Surgery forms the mainstay of management of BCC through either standard excision or Mohs micrographic surgery, and recurrence rates are lower with the latter [42]. Neck dissection is recommended in cases of cervical lymphadenopathy followed by radiotherapy or chemotherapy [43]. Although no uniform guidelines exist for treatment of advanced BCC, trials such as BOLT trial and ERIVANCE study have recommended the use of SHH pathway inhibitors such as Vismodegib or Sonidegib [44]. A previous report has described metastasis of BCC in the thyroid gland of a 45‐year‐old Caucasian male, for which hemithyroidectomy was performed; but the postoperative survival was only 12 months due to pulmonary metastasis [45]. It is, therefore, essential not to overlook the changes in the thyroid gland and the patient must be closely followed at our head and neck surgery clinic.

As there is a potential for metastasis in BCC, it is essential for healthcare professionals managing patients with such lesions to have a thorough understanding of both the diagnostic workup, clinical behavior, and therapeutic options in cases of metastatic BCC. Currently, there is a lack of consensus guidelines on managing metastatic BCC. This, along with the unpredictable nature of the disease, makes the management of metastatic BCC somewhat ad hoc and difficult. Our patient was managed in light of the recommendations made in previous studies on similar patients, and a multi‐disciplinary approach was adopted to involve all the concerned specialties. Although cervical lymphadenopathy and thyroid metastasis have previously been reported, the molecular genetic findings of our patient appear to be unique and not previously reported in metastatic BCC. This highlights the need for a full workup and sharing of data in such cases to enable more robust evidence‐based decision‐making in the diagnosis and treatment of these patients. The authors, therefore, recommend molecular testing as part of the routine workup for patients with metastatic BCC to generate evidence on the role of genetics in development/clinical course in cases of metastatic BCC. With the recent developments in novel therapeutic approaches, including targeted therapies, it has become highly imperative to unearth the complex molecular genetic characteristics of these patients to devise effective management strategies for the management of patients with metastatic BCC.

## Author Contributions

**Noora Al‐Hail:** investigation, supervision, writing – original draft, writing – review and editing. **Majid Al‐Abdulla:** writing – review and editing. **Syed Rizvi:** investigation, writing – review and editing. **Nazeeha Al‐Hayki:** investigation, writing – review and editing.

## Disclosure

Expertise: Our team comprises experts from Otolaryngology—head and neck surgery, Dermatology, oncology, Laboratory and Pathology. We have collaborated previously with a strong track record of research.

## Ethics Statement

The authors have nothing to report.

## Consent

Written informed consent was obtained from the patient for the publication of this case report, including relevant clinical details and any accompanying images. The patient's confidentiality and privacy have been maintained in accordance with local and international ethical guidelines.

## Conflicts of Interest

The authors declare no conflicts of interest.

## Data Availability

All data supporting the findings of this study are included within the article. Additional information or de‐identified data may be made available from the corresponding author upon reasonable request.
